# Papillary Thyroid Cancer-Promoting Activities of Combined Oral Contraceptive Components

**DOI:** 10.31661/gmj.v9i0.1648

**Published:** 2020-05-21

**Authors:** Mehdi Hedayati, Sadegh Rajabi, Abdolrahim Nikzamir

**Affiliations:** ^1^Cellular and Molecular Endocrine Research Center, Research Institute for Endocrine Sciences, Shahid Beheshti University of Medical Sciences, Tehran, Iran; ^2^Department of Clinical Biochemistry, School of Medicine, Shahid Beheshti University of Medical Sciences, Tehran, Iran

**Keywords:** Papillary Thyroid Cancer, Oral Contraceptives, Proliferation, Apoptosis

## Abstract

**Background:**

Thyroid cancer is more common in women at reproductive age, suggesting the relationship between its high-incidence and therapeutic use of hormonal medications, such as oral contraceptives (OCPs). The aim of this study was to identify the effect of low-dose combined OCP (LD-COC) on proliferation, apoptosis, and migration of human papillary thyroid cancer (PTC) BCPAP cell line.

**Materials and Methods:**

BCPAP cells were cultured and treated with the combination of 90nM levonorgestrel (LNG) and 20nM ethinylestradiol (EE) for 48 hours. Afterward, using 3-(4, 5-dimethylthiazol-2-yl) -2, 5-diphenyltetrazolium bromide (MTT) assay, the proliferation of the cells was measured. Apoptosis was determined by using a Caspase-3 ELISA kit. Migratory properties of combined LNG and EE were studied through wound scratch assay. The expression levels of pro-apoptotic factor *BAX*, anti-apoptotic factor *Bcl2*, and proliferation marker *Ki67* were analyzed by quantitative reverse transcriptase polymerase chain reaction (qRT-PCR) and western blotting.

**Results:**

Upon treatment with the combination of LNG and EE, proliferation and migration of BCPAP cells were significantly enhanced. However, LNG and EE remarkably inhibited apoptosis of these cells. Furthermore, treating PTC cells with combined LNG and EE caused a marked increase in the expression of *Bcl2* and *Ki67* and a considerable decrease in *BAX* levels (P˂0.05).

**Conclusion:**

Our data linked the use of COCs and the progression and aggressiveness of PTC, suggesting the role of these hormonal compounds as promoting factors for PTC tumors. Despite these observations, further investigations will be required to fully establish the pathogenic impact of these medications on PTC.

## Introduction

During the past few decades, the incidence of thyroid cancer is growing across the globe. This augmentation may affect various age and ethnic groups with an incremented risk among women less than 45 years of age [1]. Thyroid cancer is one of the rapidly increasing cancer diagnoses worldwide. Its incidence is approximately three times more prevalent in women in comparison to men [2]. Papillary thyroid cancer (PTC) is the most frequent histologic category of thyroid cancer [3]. The incidence rate of PTC among females has been estimated as 2.6 times more than males with a peak in reproductive years in females, while elevates with age in males [3,4]. This proposes that the hormonal and reproductive factors may play an important role in the incremented incidence of thyroid cancer in women. There are several lines of contradictory findings on the role of hormonal factors in increasing the rate of PTC [5-9]. Hormonal factors can be classified into exogenous and endogenous types. Oral contraceptives (OCPs) are one of the exogenous hormones used by females that are produced as single or combined preparations of synthetic estrogens and progestogens [10]. Combined OCPs (COCs) are the most commonly used contraceptives. There are differences in the types of combined pills in different parts of the world. However, low-dose (LD) contraceptives containing levonorgestrel (LNG) and ethinylestradiol (EE) are the most commonly used formulations in Iran and most European countries [11,12]. The use of OCPs in some cancers is contraindicated to prevent their adverse effects. For example, in the case of breast cancer, it is thought that using contraceptives may not be a cancer initiator, but it may act as promoting factor for tumor growth [13,14]. The World Health Organization Medical Eligibility Criteria for Contraceptive Use categorized the breast cancer cases (within five years of initial diagnosis) as category four, which means that hormonal methods should be contraindicated [15]. There are multiple previous evidences that indicate the expression of estrogen and progesterone receptors (ER and PR) in PTC [16-18]. Also, the expression of these receptors in PTC samples has been shown to significantly higher than those in controls [19,20]. These results could propose that steroid hormones can bind these receptors and exert their growth-promoting effects in this type of cancer. There are very limited data concerning the effect of estrogen and progesterone on the progression of PTC. Nonetheless, there is no previous data investigating the role of OCP components as promoting factors for PTC patients. In this light, this study aimed to explore the combined effects of LNG and EE (as LD-COCs) on the promotion and progression of BCPAP cell line.

## Materials and methods

###  Cell Culture

BCPAP (as human PTC cell line) were purchased from Pasteur Institute (Tehran, Iran). Cells were cultured in Roswell Park Memorial Institute (RPMI) 1640 medium, which was supplemented with 10% fetal bovine serum (FBS, Gibco, Germany) and 1% penicillin/streptomycin (Biosera, England), as described by Zeng *et al* [21]. When cell confluency reached 60–70%, the medium was changed to phenol red-free RPMI 1640 medium supplemented with 10 % charcoal-stripped FBS(Sigma Chemical, St. Louis, MO) for 24 h before treatment with the combination of EE and LNG (Aburaihan Pharmaceutical Co., Tehran, Iran).

###  Cell Proliferation and Apoptosis Assay

The proliferation of the cells was assayed according to the protocol described by Rajoria *et al*. with slight modifications [22]. Briefly, BCPAP cells were harvested using 1% trypsin (Biosera, England) and seeded at a density of 6×103 cells per well in 96-well culture plates and allowed to adhere for approximately 16-20 hours. After that, the cells were washed with phosphate-buffered saline (PBS) and starved for 12 hours. The starvation medium was prepared by dissolving 1% penicillin/streptomycin in phenol red-free RPMI 1640 medium. Subsequently, the test group cells were treated with the combination of 20 nM EE and 90 nM LNG, and control cells left untreated for 24, 48, and 72 hours. Concentrations of both EE and LNG were calculated based on the amount of both compounds available in LD-OCP tablets dissolved in 5 liters of blood. Both EE and LNG were dissolved in ethanol to prepare above mentioned concentrations. Dimethylthiazol diphenyltetrazolium bromide (MTT) assay was utilized to quantify the proliferation of BCPAP cells based on cell viability. After the end of treatment periods, 100 µl MTT solutions was added to each well and incubated for 3 hours. Then, these solutions were replaced by 100 µl Dimethyl sulfoxide (DMSO), and subsequently, the absorbances of the wells were measured at 570 nm. The percentage of viable BCPAP cells was calculated by dividing the absorbance of treated cells by the absorbance of untreated cells at 570 nm and multiplied by 100. Apoptosis was detected by measuring Caspase-3 level, using a Caspase-3 ELISA kit (Invitrogen, USA) according to instructional guides provided by manufacturers.

###  Wound Healing Assay

Migratory capacity of BCPAP cells following treatments was also evaluated by in vitro as previously demonstrated [23]. Briefly, 5×105 cells were seeded in a 6-well plate and allowed to adhere and grow to 60–70%. Then, four horizontal wounds were created per well using a 200 µl sterile pipette tip in the center of each well. Subsequently, cell debris was washed, and the wells were treated with the combination of 20 nM EE and 90 nM LNG for 48h. One vertical line was made to assure the visualization of wounds at the same point. After the treatment, the wound closure photographs and the empty scratched areas were captured and measured by using Optika Vision Pro version 4.4, at 0h, 12h, 24h and 48h of post-treatment.

###  Real-Time Polymerase Chain Reaction (PCR)

Based on results of MTT assay, after treating the test cells with the combination of 20 nM EE and 90 nM LNG, and control cells with the normal medium for 48h, the total RNA was isolated using RNeasy Mini, RNA isolation kit (Qiagen, Germany) according to manufacturer’s instructions. RNA concentration was measured by Nanodrop 2000c spectrophotometer (Thermo Scientific, USA), and cDNA were synthesized by cDNA Synthesis Kit (Bio FACT, Daejeon, South Korea). The changes in mRNA expression of *MKI67*, *BAX*, *Bcl2* genes and Beta-2-microglobulin (β2M), as internal control, were estimated by quantitative reverse transcriptase PCR (qRT-PCR) in a rotor gene 6000 Corbett (Corbett Research, Sydney, Australia) detection system SYBR GREEN® (nonspecific DNA-binding factors). The primer sequences are provided in Table-1. Alterations of gene expression of *KI67*, *BAX,* and *Bcl2* compared with β2M were normalized by LinReg software (Linreg version 2012.1, Amsterdam, the Netherlands). Expression levels of the mentioned gens were quantified by REST program (Relative Expression Software Tool, Qiagen, Germany).

###  Western Blotting

 According to the results obtained from the MTT assay, combination of 20 nM EE and 90 nM LNG was chosen to treat cells of the test group, and control cells were treated with normal medium. After the 48h, treated cells were harvested using 1% trypsin, washed with PBS, and lysed by the radioimmunoprecipitation assay (RIPA) buffer 70 mM Tris-HCl (pH 7.4), 100 mM NaCl, 0.5% sodium deoxycholate, 0.1% SDS, 1.5 µM Pefabloc), and incubated on ice for 30 minutes with shaking. Afterward, the cell lysates were centrifuged at 15000 rpm for 20 min at 4°C, and the supernatant was collected. Subsequently, 40 μg of protein was separated by SDS-PAGE followed by transferring the gel to nitrocellulose membranes. After blocking by 5% skimmed milk in TBST (200 mM Tris–HCl, pH 7.4, 100 mM NaCl, and 0.05% Tween-20) for 3h on a shaker at room temperature, the membranes were incubated with primary antibodies including Ki67, BAX, Bcl2, and GAPDH (Santa Cruz Biotechnology, Santa Cruz, CA) overnight on a shaker at 4°C in TBST. After 16-18h, membranes were washed three times with TBST and incubated with the corresponding horseradish peroxidase conjugated secondary antibody, for 1h at room temperature in TBST containing 1% milk. 3,3′-diaminobenzidine (DAB) solution (Sigma Chemical, St. Louis, MO) and 0.3% hydrogen peroxide (Merck, Germany) were used as the substrate to develop membranes, the reaction was stopped by washing the blot with water, and the image of the blot was acquired. Finally, using Image J software, the blots were semi-quantified.

###  Statistical Analysis

 All the data were presented as mean ± SD for at least three replicates. Statistical significance and differences between groups were examined using Student’s t-test, and Mann–Whitney U test. A P˂ 0.05 was considered as significant level. All data were statistically analyzed using GraphPad Prism 7.

## Results

###  Combined Effects of EE and LNG On Proliferation and Apoptosis of BCPAP Cells

**Figure 1:**
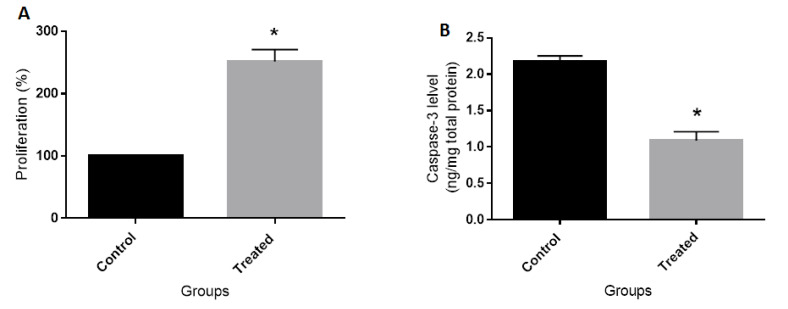
Effects of combined LNG and EE on proliferation and apoptosis of BCPAP cells. **A**: Cell proliferation was examined using the MTT assay. **B**: Apoptosis was detected by measuring Caspase-3 level in treated and control groups. The data are expressed as mean ± SD and represent one of at least three separate experiments. *P˂0.01 vs. control.

 As shown in Figure-1a, the combination of EE and LNG for 48h significantly increased the proliferation of BCPAP cells compared with the corresponding control cells. We also treated the cells with a range of concentrations of EE (0.2-2000nM) and LNG (0.9-9000nM) for 24, 48, and 72 hours (data not shown) to test the effects of different combined concentrations of the drugs on the proliferation of BCPAP cells. Interestingly, we found that only the combination of 20 nM EE and 90 nM LNG for 48h could exert remarkable proliferative effects on BCPAP cells. Accordingly, all the following tests were performed using these optimum concentrations and time. Treating the cells with combination of 20 nM EE and 90 nM LNG for 48h significantly decreased the level of Caspase-3 in comparison to untreated cells (Figure-1b).

###  Combination of EE and LNG Enhances Migration of BCPAP Cells

**Figure 2:**
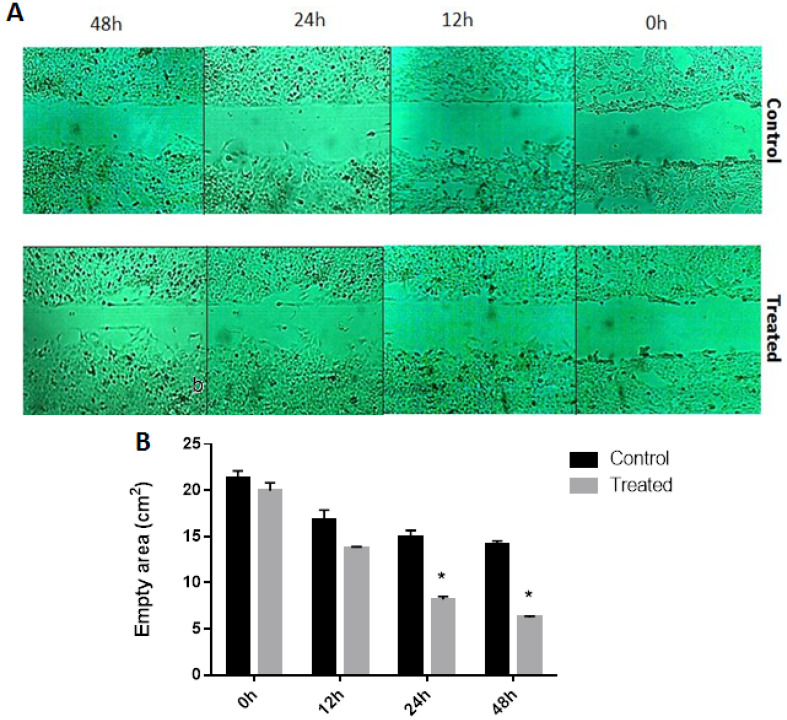
Migratory properties of combined treatment BCPAP cells by LNG and EE. (a)ethinylestradiol. **A**: The subconfluent BCPAP cells were scratched with a sterile pipette tip and then treated with the combination of 20nM EE and 90nM LNG for 48 h. Images of wound closure were captured at 0 h, 24 h and 48 h of post-treatment and wound areas were measured at each time point. **B**: Data are expressed as cell migration area (cm2) compared with control (mean ± SD). *P<0.05 vs. control.

The migration of tumor cells away from the primary site, a process called tumor invasion, has been previously demonstrated as the first and pivotal step in cancer metastasis; hence, it has been proven that invasion and metastasis are two hallmarks of tumor malignancy [24]. Therefore, we aimed to conduct a monolayer wound healing assay to analyze the migratory effects of combined 20 nM EE and 90 nM LNG on BCPAP cells. As depicted in Figure-2a, we observed that the combination of OCP components time-dependently enhanced the migration of the BCPAP cells toward the middle of the scratches. According to data shown in Figure-2b, the empty scratched areas were also significantly time-dependently decreased in treated cells compared with control cells.

###  Gene Expressions Following Ethinylestradiol and LNG Exposure

**Figure 3:**
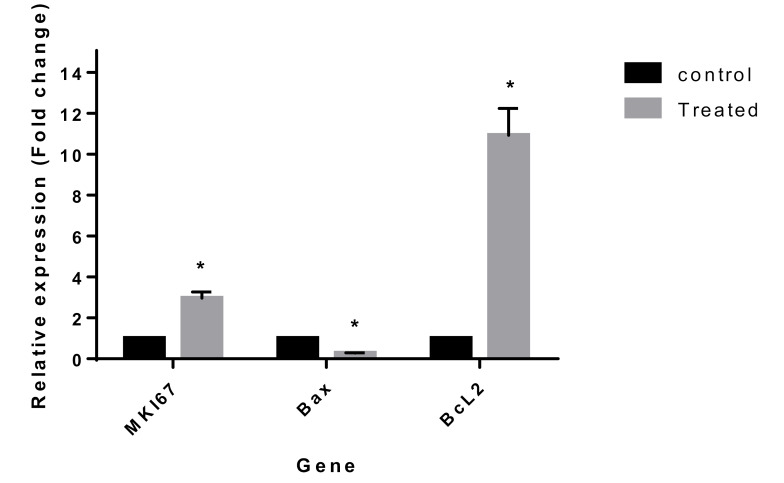
Effects of combined LNG and EE treatment of BCPAP cell line on the expression levels of *MKI67*, *BAX,* and *Bcl2* genes. The mRNA expressions were analyzed by real-time PCR. The results are representative of at least three separate experiments. *P˂0.05 vs. control.

It is now widely accepted that *MKI67, BAX, and Bcl2* are markers of proliferation, pro-apoptotic, anti-apoptotic genes, respectively [25,26]. In order to provide direct molecular evidence to confirm the prolifratory and anti-apoptotic properties of the OCP components in PTC cell line, we treated the test cells with the combination of 20 nM EE and 90 nM LNG and the controls with normal medium for 48h. Subsequently, the mRNAs expression of *MKI67*, *BAX,* and *Bcl2* were measured. The results showed that the combined EE and LNG treatment considerably induced the expression of *MKI67* and *Bcl2 *and markedly reduced *BAX* mRNA expression levels as compared with untreated control cells (Figure-3).

###  Protein Expressions Following Treatment with the Combined Ethinylestradiol and LNG

**Figure 4:**
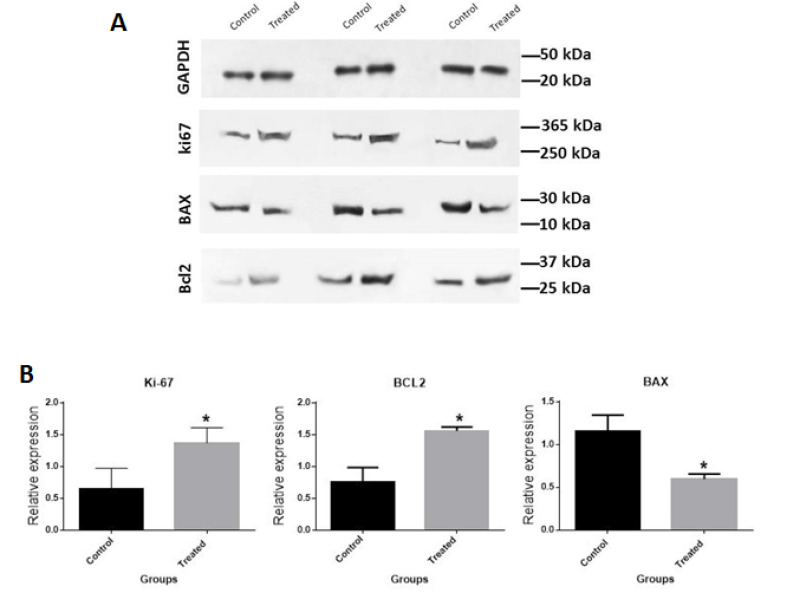
**A**: Effects of combined LNG and EE treatment on the expressions Ki67, BAX, and Bcl2 proteins in the BCPAP cells. **B**: Treating cells with EE and LNG remarkably inhibited BAX expression; however, it caused a marked increase in the expression of Ki67 and Bcl2 proteins when compared with untreated control cells. *P˂0.05 vs. control.

 To further confirmation of the proliferative and anti-apoptotic effects of our tested OCP components, we used western blot analysis. Consistent with the Real time-PCR results, EE and LNG combination considerably upregulated Ki67 and Bcl2 and remarkably downregulated Bax protein expression levels (Figure-4). These results provide evident data to verify proliferation, promoting and apoptosis inhibiting effects of our tested OCP compounds at the protein level.

## Discussion

Epidemiological data proposed that well-differentiated thyroid carcinomas such as PTCs predominately occur in females who are post-pubertal and pre-menopausal, and OCP use by fertile women is accompanied by a moderately higher risk of thyroid cancer, although there are some controversies in this regard [27]. The expression levels of estrogen receptor (ER), especially ERα, have been shown to be high in female thyroid cancer patients using OCPs and in well-differentiated cancer cases [28]. Kawabata *et al*. indicated that estrogens might be involved in the physiologic and pathologic events of thyroid gland, the processes that occur via ERα. They also asserted that neoplastic alterations in thyroid, which affect pre-menopausal women, considerably highlight the role of these receptors in tumor development and progression [29]. Moreover, it appears that promoting effects of estradiol on thyroid tumorigenesis take place due to activation of thyrotrophs in the pituitary or estradiol-ER interaction in the thyroid [30]. An augmented expression of ERs has been reported in PTC cell lines such as BCPAP, KAT5 [20,31,32]. Although thyroid cancer commonly affects women in fertile age and PTC is the most frequent type among women, there is no previous study examining the roles of COC components in thyroid cancer promotion. This possibility prompted us to assess promoting effects of combined LNG and EE, LD-COC components, on PTC cells. Our functional study data revealed that the combination of LNG and EE significantly induced the proliferation and reduced apoptosis of BCPAP cells as compared with control cells. Moreover, this combination treatment caused a marked increase in the migratory characteristic of these cells compared with untreated control cells. The results of Real time-PCR and western blotting analyses also showed remarkable increment in the expression levels of *Ki67 *(nuclear marker of proliferation), and *Bcl2* (anti-apoptotic marker), and a marked reduction in the expression of *BAX* (pro-apoptotic marker), at protein and mRNA levels in comparison to controls. Our obtained data are in agreement with the results of several previous studies, which they have investigated the promoting role of estrogen derivatives in the thyroid cancer progression. Vivacqua *et al*. demonstrated that 17β-estradiol (E2) could stimulate the expression of some genes implicated in cell cycle progression and the proliferation of human follicular and anaplastic thyroid cancer cells; even in the cell line that does not express ERs. They linked these effects to G protein-coupled receptor 30 (GPR30) and the mitogen-activated protein kinase (MAPK) pathway activation [33]. Kumar *et al*. attributed the E2 -induced PTC and follicular thyroid cancer cell proliferation to ERα and ERβ transcriptional and non-genomic signaling pathways by involving ERK1/2 phosphorylation, which is triggered through membrane-bound GPR30 [34]. Additionally, they reported that the proliferative actions of E2 in PTC cell line (KAT5) are mainly mediated by ERα [34]. The thyroid gland has been shown to have the capability of estrogen synthesis and responsiveness, the two potentials that seem to be enhanced during tumor development [33]. Treating PTC cells with E2 has been reported to cause an increased ERα expression, cell proliferation, and reduction of *BAX* level in the mitochondria [35]. It appears that the enhanced proliferation, and inhibited apoptosis, which were observed in our study, may be due to increased expression of ERα in BCPAP cells. Meanwhile, Zeng *et al*. asserted that the function of ERα dominates that of ERβ in PTC cells. Some studies have suggested that the promoting effects of E2 on PTC cells may be related to apoptotic pathway inhibition through up-regulation of anti-apoptotic factors (such as *Bcl2* and *Bcl-xL*) as well as down-regulation of pro-apoptotic factor *Bax* [21,36]. However, as we observed in our study, high antigen *Ki67* expression levels were strongly associated with tumor cell proliferation. *Ki67*, as a proliferation marker, is a well-known and established prognostic and predictive biomarker in different type of cancers [37], which its expression is correlated with metastasis and clinical stage of tumors [38]. Our migration study also provided evidence to confirm the presence of a positive correlation between the increased levels of *Ki67* and enhanced migratory characteristics of BCPAP cells. Rajoria *et al*. also found that E2 treatment could markedly increase migration, invasion, and adhesive properties of normal and malignant thyroid cells, which have been suggested to be modulated by β-catenin [22]. Tumorigenesis properties of progesterone and progestins have been reported in some studies on breast cancer cells. Lee *et al*. indicated that medroxyprogesterone acetate and progesterone treatment could non-significantly increase tumor formation and burden and significant cell proliferation and angiogenesis, the process that is pivotal for metastasis, in rat models of breast cancer. Moreover, they showed an approximately 2-fold increase in the growth of spheroid structures formed from breast cancer cell line T47D following the treatment with combined estradiol and progesterone [39]. Moore *et al*. provided an evidence to reveal the anti-apoptotic features of progestin in two breast cancer cell lines, one with a moderate expression of PR, and one with no apparent PR expression, suggesting a receptor-independent activity of these steroids [40]. More interestingly, Jeng *et al*. have suggested estrogenic properties for synthetic progestins used in OCPs in stimulating cancer growth. They have claimed that pro-proliferative activities of these OCP components are mediated by activation of ER, but not PR [41]. Another study demonstrated that progesterone exerts its anti-apoptotic effects on breast cancer cells via binding to another type of receptors called membrane PRs [42]. Taken together, regardless of the fact which type of receptor was involved in the growth-promotion effects of our tested estrogen and progestin, it appears that these steroids act synergistically in inducing proliferation and migration and inhibiting cell death in PTC cells and this may exert more detrimental influences on the progression and aggressiveness of this type of thyroid cancer.

## Conclusion

 As a whole, our findings proposed a strong association between estrogen and progestin components of COCs and thyroid cancer progression as verified by their effects on proliferation, cell death, and migration of BCPAP cells. Although the present results revealed a growth-promoting role for COCs in PTC and may suggest the precautions for using these hormonal medications in the PTC patients, we are still very much in the early years of considering a contraindication for COCs use in these patients. Indeed, our data highlighted or underscored the need for detailed studies to dissect the role of these steroids in the progression and aggressiveness of PTC tumors.

## Acknowledgment

 This paper has been extracted from the Ph.D. thesis of Sadegh Rajabi and was supported by grant number: 96007 from Endocrine Research Center, Research Institute for Endocrine Sciences, Shahid Beheshti University of Medical Sciences, Tehran, Iran.

## Conflict of Interest

 The authors have no conflict of interest to declare.

**Table 1:** Primer Sequences Used in This Study

**Gene name**	**Forward primer**	**Reverse primer**
*BAX*	CCCTTTTGCTTCAGGGTTTCAT	ACTCGCTCAGCTTCTTGGTG
*Bcl2*	CTGTGGATGACTGAGTACCT	GCCAGGAGAAATCAAACAGAG
*MKI67*	GCTACTCCAAAGAAGCCTGTG	AAGTTGTTGAGCACTCTGTAGG
*β 2 M*	TGTCTTTCAGCAAGGACTGGT	TGCTTACATGTCTCGATCCCAC

## References

[1] Khaled H, Al Lahloubi N, Rashad N (2016). A review on thyroid cancer during pregnancy: Multitasking is required. J Adv Res.

[2] Rahbari R, Zhang L, Kebebew E (2010). Thyroid cancer gender disparity. Future Oncol.

[3] Wang P, Lv L, Qi F, Qiu F (2015). Increased risk of papillary thyroid cancer related to hormonal factors in women. Tumour Biol.

[4] Kilfoy BA, Devesa SS, Ward MH, Zhang Y, Rosenberg PS (2009). Gender is an age-specific effect modifier for papillary cancers of the thyroid gland. Cancer Epidemiol Biomarkers Prev.

[5] Horn-Ross PL, Canchola AJ, Ma H, Reynolds P, Bernstein L. Hormonal factors and the risk of papillary thyroid cancer in the California Teachers Study cohort. Cancer Epidemiol Biomarkers Prev. 2011. 10.1158/1055-9965.EPI-11-0381 PMC3288117 21791618

[6] Rossing MA, Voigt LF, Wicklund KG, Williams M, Daling JR (1998). Use of exogenous hormones and risk of papillary thyroid cancer (Washington, United States). Cancer Causes Control.

[7] Sakoda LC, Horn-Ross PL (2002). Reproductive and menstrual history and papillary thyroid cancer risk: the San Francisco Bay Area thyroid cancer study. Cancer Epidemiol Biomarkers Prev.

[8] Rossing MA, Voigt LF, Wicklund KG, Daling JR (2000). Reproductive factors and risk of papillary thyroid cancer in women. Am J Epidemiol.

[9] Kabat GC, Kim MY, Wactawski-Wende J, Lane D, Wassertheil-Smoller S, Rohan TE (2012). Menstrual and reproductive factors, exogenous hormone use, and risk of thyroid carcinoma in postmenopausal women. Cancer Causes Control.

[10] Bitzer J (2013). Oral contraceptives in adolescent women. Best Pract Res Clin Endocrinol Metab.

[11] Brynhildsen J (2014). Combined hormonal contraceptives: prescribing patterns, compliance, and benefits versus risks. Ther Adv Drug Saf.

[12] Pakgohar M, Malekian S (2015). Impact of Oral Contraceptive Pills (LDs) and Condoms on Women’s Sexual Function: A Prospective Study in Iran. Nurs Heal.

[13] Veljkovic M, Veljkovic S (2010). [The risk of breast cervical, endometrial and ovarian cancer in oral contraceptive users]. Med Pregl.

[14] Colditz GA (2007). Decline in breast cancer incidence due to removal of promoter: combination estrogen plus progestin. Breast Cancer Res.

[15] Kubba A (2003). Breast cancer and the pill. Journal of the Royal Society of Medicine.

[16] Eldien MMS, Abdou AG, Rageh T, Abdelrazek E, Elkholy E (2017). Immunohistochemical expression of ER-alpha and PR in papillary thyroid carcinoma. Ecancermedicalscience.

[17] Jalali-Nadoushan MR, Amirtouri R, Davati A, Askari S, Siadati S (2016). Expression of estrogen and progesterone receptors in papillary thyroid carcinoma. Caspian J Intern Med.

[18] Sturniolo G, Zafon C, Moleti M, Castellvi J, Vermiglio F, Mesa J (2016). Immunohistochemical Expression of Estrogen Receptor-alpha and Progesterone Receptor in Patients with Papillary Thyroid Cancer. Eur Thyroid J.

[19] Chen D, Qi W, Zhang P, Guan H, Wang L (2015). Expression of the estrogen receptor alpha, progesterone receptor and epidermal growth factor receptor in papillary thyroid carcinoma tissues. Oncol Lett.

[20] Di Vito M, De Santis E, Perrone GA, Mari E, Giordano MC (2011). Overexpression of estrogen receptor-alpha in human papillary thyroid carcinomas studied by laser- capture microdissection and molecular biology. Cancer Sci.

[21] Zeng Q, Chen GG, Vlantis AC, van Hasselt CA (2007). Oestrogen mediates the growth of human thyroid carcinoma cells via an oestrogen receptor-ERK pathway. Cell Prolif.

[22] Rajoria S, Suriano R, Shanmugam A, Wilson YL, Schantz SP (2010). Metastatic phenotype is regulated by estrogen in thyroid cells. Thyroid.

[23] Khan S, Shukla S, Sinha S, Lakra AD, Bora HK, Meeran SM (2015). Centchroman suppresses breast cancer metastasis by reversing epithelial-mesenchymal transition via downregulation of HER2/ERK1/2/MMP-9 signaling. Int J Biochem Cell Biol.

[24] Clark AG, Vignjevic DM (2015). Modes of cancer cell invasion and the role of the microenvironment. Curr Opin Cell Biol.

[25] Sales Gil R, Vagnarelli P (2018). Ki-67: More Hidden behind a ‘Classic Proliferation Marker’. Trends Biochem Sci.

[26] Singh L, Pushker N, Saini N, Sen S, Sharma A (2015). Expression of pro-apoptotic Bax and anti-apoptotic Bcl-2 proteins in human retinoblastoma. Clin Exp Ophthalmol.

[27] Ceresini G, Morganti S, Graiani V, Saccani M, Milli B (2006). Estrogen receptor (ER)-beta, but not ER-alpha, is present in thyroid vessels: immunohistochemical evaluations in multinodular goiter and papillary thyroid carcinoma. Thyroid.

[28] Bufalo N.E., Campos A.H., Rocha A.G., Cunha L.L., Pimentel G.D., Batista F.A. et al. ERα IMMUNOSTAINING: AN AUXILIARY DIAGNOSTIC TOOL OF FOLLICULAR THYROID LESIONS. 15TH INTERNATIONAL THYROID CONGRESS PROGRAM AND MEETING ABSTRACTS; Florida, USA. Thyroid: Mary Ann Liebert, Inc.; 2015.

[29] Kawabata W, Suzuki T, Moriya T, Fujimori K, Naganuma H (2003). Estrogen receptors (alpha and beta) and 17beta-hydroxysteroid dehydrogenase type 1 and 2 in thyroid disorders: possible in situ estrogen synthesis and actions. Mod Pathol.

[30] Mori M, Naito M, Watanabe H, Takeichi N, Dohi K, Ito A (1990). Effects of sex difference, gonadectomy, and estrogen on N-methyl-N-nitrosourea induced rat thyroid tumors. Cancer Res.

[31] Dong W, Zhang H, Li J, Guan H, He L (2013). Estrogen Induces Metastatic Potential of Papillary Thyroid Cancer Cells through Estrogen Receptor alpha and beta. Int J Endocrinol.

[32] Rajoria S, Parmar P, Schantz S, Schaefer S, Chaudhuri D (2008). Significance of estrogen receptor in thyroid cancer: Molecular link of epidemiologic observations. Cancer Res.

[33] Vivacqua A, Bonofiglio D, Albanito L, Madeo A, Rago V (2006). 17beta-estradiol, genistein, and 4-hydroxytamoxifen induce the proliferation of thyroid cancer cells through the g protein-coupled receptor GPR30. Mol Pharmacol.

[34] Kumar A, Klinge CM, Goldstein RE (2010). Estradiol-induced proliferation of papillary and follicular thyroid cancer cells is mediated by estrogen receptors alpha and beta. Int J Oncol.

[35] Zeng Q, Chen G, Vlantis A, Tse G, van Hasselt C (2008). The contributions of oestrogen receptor isoforms to the development of papillary and anaplastic thyroid carcinomas. J Pathol.

[36] Lee ML, Chen GG, Vlantis AC, Tse GM, Leung BC, van Hasselt CA (2005). Induction of thyroid papillary carcinoma cell proliferation by estrogen is associated with an altered expression of Bcl-xL. Cancer J.

[37] Warth A, Cortis J, Soltermann A, Meister M, Budczies J (2014). Tumour cell proliferation (Ki-67) in non-small cell lung cancer: a critical reappraisal of its prognostic role. Br J Cancer.

[38] Li LT, Jiang G, Chen Q, Zheng JN (2015). Ki67 is a promising molecular target in the diagnosis of cancer (review). Mol Med Rep.

[39] Lee O, Choi MR, Christov K, Ivancic D, Khan SA (2016). Progesterone receptor antagonism inhibits progestogen-related carcinogenesis and suppresses tumor cell proliferation. Cancer Lett.

[40] Moore MR, Spence JB, Kiningham KK, Dillon JL (2006). Progestin inhibition of cell death in human breast cancer cell lines. J Steroid Biochem Mol Biol.

[41] Jeng MH, Parker CJ, Jordan VC (1992). Estrogenic potential of progestins in oral contraceptives to stimulate human breast cancer cell proliferation. Cancer Res.

[42] Dressing GE, Alyea R, Pang Y, Thomas P (2012). Membrane progesterone receptors (mPRs) mediate progestin induced antimorbidity in breast cancer cells and are expressed in human breast tumors. Horm Cancer.

